# Cross-domain information fusion and personalized recommendation in artificial intelligence recommendation system based on mathematical matrix decomposition

**DOI:** 10.1038/s41598-024-57240-6

**Published:** 2024-04-03

**Authors:** Xiaoyan Meng

**Affiliations:** 1 Inner Mongolia Youth College of Political Science of Inner Mongolia Normal University, Hohhot, 010051 China; 2 Youth College of Political Science of Inner Mongolia Normal University, Hohhot, 010051 China; 3 Inner Mongolia Youth League School, Hohhtot, 010051, China

**Keywords:** Personalized recommendation, Matrix factorization algorithm, Cross-domain information fusion, Data sparsity, Collaborative filtering algorithm, Mathematics and computing, Applied mathematics, Computational science, Computer science, Information technology

## Abstract

Given the challenges of inter-domain information fusion and data sparsity in collaborative filtering algorithms, this paper proposes a cross-domain information fusion matrix decomposition algorithm to enhance the accuracy of personalized recommendations in artificial intelligence recommendation systems. The study begins by collecting Douban movie rating data and social network information. To ensure data integrity, Levenshtein distance detection is employed to remove duplicate scores, while natural language processing technology is utilized to extract keywords and topic information from social texts. Additionally, graph convolutional networks are utilized to convert user relationships into feature vectors, and a unique thermal coding method is used to convert discrete user and movie information into binary matrices. To prevent overfitting, the Ridge regularization method is introduced to gradually optimize potential feature vectors. Weighted average and feature connection techniques are then applied to integrate features from different fields. Moreover, the paper combines the item-based collaborative filtering algorithm with merged user characteristics to generate personalized recommendation lists.In the experimental stage, the paper conducts cross-domain information fusion optimization on four mainstream mathematical matrix decomposition algorithms: alternating least squares method, non-negative matrix decomposition, singular value decomposition, and latent factor model (LFM). It compares these algorithms with the non-fused approach. The results indicate a significant improvement in score accuracy, with mean absolute error and root mean squared error reduced by 12.8% and 13.2% respectively across the four algorithms. Additionally, when k = 10, the average F1 score reaches 0.97, and the ranking accuracy coverage of the LFM algorithm increases by 54.2%. Overall, the mathematical matrix decomposition algorithm combined with cross-domain information fusion demonstrates clear advantages in accuracy, prediction performance, recommendation diversity, and ranking quality, and improves the accuracy and diversity of the recommendation system. By effectively addressing collaborative filtering challenges through the integration of diverse techniques, it significantly surpasses traditional models in recommendation accuracy and variety.

## Introduction

The rapid development of artificial intelligence has made the application of recommendation system in various fields increasingly extensive, providing users with a more personalized experience. In collaborative filtering algorithms, inter-domain information fusion^1,2^ and data sparsity^3,4^ have always been important factors restricting the performance of recommendation systems. In order to overcome these problems, this paper combines cross-domain information with matrix decomposition algorithm to achieve more accurate personalized recommendation in artificial intelligence recommendation system.

The field of personalized recommendations has been extensively studied in the past and multifaceted solutions have been explored. In order to introduce the correlation between user preference and time into personalized recommendation technology, Cui Zhihua et al.^5^ proposed a recommendation model TCCF (time-constrained Correlation Factor) based on time correlation coefficient and improved cuckoo search K-means. Compared to the MCoC (Model of Concept of Coherence) model, the accuracy of the model is improved by about 5.2%. In order to simulate the interaction between patient covariate and treatment effect to provide personalized treatment recommendations, Katzman Jared et al.^6^ proposed DeepSurv, which combines Cox proportional risk model with deep neural network to achieve accurate prediction ability. In order to fully explore the potential structural features of users and items in the Heterogeneous Information network-based recommendation method, Shi Chuan et al.^7^ proposed a HERec model based on heterogeneous Network embedding and Heterogeneous Information Network (HIN). He achieved this by combining optimization of the extended MF (Matrix Factorization) model and fusion function. In order to realize customized optimization of recommendation tasks, He Xiangnan et al.^8^ proposed a Neural network model NAIS (Neural Attentive Item Similarity) based on CF (Collaborative Filtering). This can effectively distinguish which historical items in the user profile are more important to the prediction. In order to capture the complex decision-making process of users, Xue Feng et al.^9^ proposed a solution in project-based collaborative filtering recommendation. While modeling the second-order interaction between the two items, he used a nonlinear neural network to consider the interactions between all interacting pairs of items. The above method is suitable for effective personalized recommendation, but due to the influence of data noise and missing values, the accuracy of recommendation may be reduced.

In previous studies, many scholars have introduced mathematical matrix decomposition technology into the implementation of recommendation system. In order to build a crowdsourced task recommendation system, Shu Jiangang et al.^10^ proposed a key derivation method based on matrix decomposition, which effectively ensured the user accountability and retract mechanism and improves the efficiency of the system. In order to develop recommendation systems in Mashup applications, Yao Lina et al.^11^ designed a latent variable model using mathematical probability matrix decomposition and implicit correlation regularization to address the accuracy and diversity of recommendations. In order to improve the performance of GeoMF (Geographical Matrix Factorization) model in place recommendation system, Lian Defu et al.^12^ combined two-dimensional kernel density estimation method with geographic modeling and matrix decomposition based on implicit feedback, thus achieving higher scalability. In order to improve the accuracy of industrial recommendation systems, Liu Hai et al.^13^ proposed an efficient deep matrix decomposition method with retrospective feature learning, which had broad industrial application prospects. Based on user purchase history and product rating, Sallam Rouhia et al.^14^ successfully built a product recommendation system through mathematical matrix decomposition theory and project-based collaborative filtering and singular value decomposition based collaborative filtering methods. In order to use implicit feedback in the recommendation system, Liu Yu et al.^15^ proposed an autoencoder deep hybrid recommendation system framework based on mathematical matrix decomposition, and built a hybrid recommendation system by integrating user side and commodity side information to improve the system performance. In order to perform unconstrained non-negative potential factor analysis of the HiDS (high-dimensional signature) in Unconstrained Nonnegative Latent Factor Analysis (UNLFA), Luo Xin et al.^16^ innovatively transferred non-negative constraints from decision parameters to output LFs (Latent Factors). He connected them through unit element-dependent mapping functions to maintain the important characteristics of high-dimensional sparse matrices in the recommendation system. In order to automatically recommend products to users, Yi Baolin et al.^17^ built a deep matrix decomposition recommendation system based on implicit feedback embedding. By abstracting the interaction behavior between users and projects into the form of a matrix, matrix decomposition technology can help the system understand and capture the potential relationships and patterns hidden behind the data, so as to provide more detailed and personalized recommendations for the recommendation system^18,19^. However, in the field of collaborative filtering algorithms, there are still problems of cross-domain information fusion and data sparsity in the process of mathematical matrix decomposition. The primary challenges in the study include addressing data sparsity and achieving effective cross-domain information fusion. To tackle these issues, a matrix decomposition algorithm with regularization methods was adopted, integrating features from various data sources. In this research, the main challenges are further improving accuracy and the quality of recommendations.

In this paper, a mathematical matrix decomposition algorithm is used to solve the problem of interdomain information fusion and data sparsity in recommendation systems. By processing Douban movie rating data and its social network information, this paper deals with data duplication and missing by combining Levenshtein distance^20,21^ and KNN (K-nearest neighbors) interpolation^22,23^. It constructs user topics and keywords by means of word segmentation, part-of-speech tagging, morphology reduction and word frequency analysis. In this paper, user-movie score matrix and user-social network relationship matrix are constructed and decomposed by Ridge regularization method^24,25^. By means of weighted average and feature connection, features from different fields are integrated^26,27^ to generate personalized recommendation lists. In the experimental stage, the mathematical matrix decomposition algorithm optimized by cross-domain information fusion is compared with traditional algorithms such as ALS^28^, SVD^29^, NMF^30^ and LFM^31^. The results show that after cross-domain information fusion optimization, the MAE mean value of the four algorithms was reduced by 13.9%, 13.8%, 13.7% and 12.8%, and the RMSE mean value was reduced by 13.2%, 13.9%, 13.8% and 13.4%, respectively. When K value increased to 100, F1 score remained above 0.76; the RAC average of LFM algorithm under different K values reached 0.83, which was 54.2% higher than that before cross-domain information fusion. The results show that the mathematical matrix decomposition optimization algorithm with cross-domain information fusion has significant advantages over the traditional methods in scoring accuracy, ranking accuracy, diversity and ranking quality.

## Data collection and preprocessing

**Figure 1:**
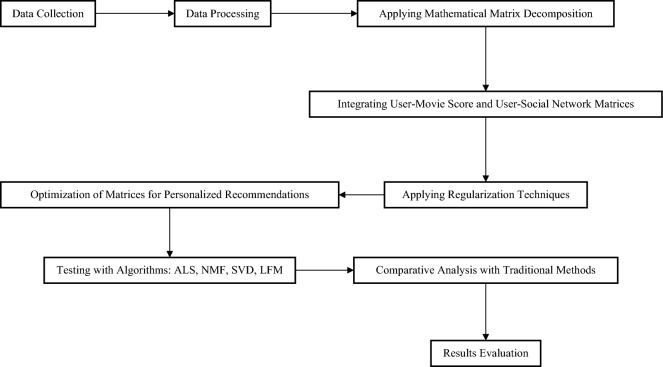
Overview of the methodology process.

The flowchart shown in Fig. 1 starts with "Data Collection" followed by "Data Processing". The processed data is then analyzed through "Mathematical Matrix Decomposition", and matrices of "User-Movie Ratings" and "User-Social Network" are integrated. Next, "Regularization Techniques" are applied to optimize these matrices in preparation for "Personalized Recommendations". The optimized matrices are tested using various algorithms (ALS, NMF, SVD, LFM) and compared with "Traditional Methods". Finally, the results are evaluated in "Results Evaluation", completing the process. The flowchart details how the accuracy and quality of recommendation systems are enhanced by integrating data from different domains and optimizing algorithms.

In our study, we collected extensive data from the Douban network platform, which included user ratings, movie information, user social network data, and user comments on social media platforms. The user rating data comprised 120,000 entries with user IDs, movie IDs, rating scores, and timestamps. Movie information data covered 2,500 movie IDs, including movie names, genres, descriptions, durations (ranging from 90 to 150 min), release dates (spanning 2000–2023), shooting dates, language types, lists of actors (averaging 15 per movie), and lists of directors. The user social network information contained records for 30,000 users, including user IDs, gender, age (ranging from 18 to 65 years), and group membership lists (with each user belonging to an average of 5 groups).

The user social platform speech data encompassed 50,000 entries from Douban topics, detailing individual posts and comments. In this dataset, we observed that 40% of the movies were categorized as drama, 25% as action, and 15% as comedy, with the remaining 20% spread across various genres. The average user rating was 3.5 out of 5, and 60% of the users actively participated in social groups related to movie discussions. Notably, we found a correlation between the frequency of social media activity and the diversity of movie genres rated by users.

This article uses Levenshtein distance to measure the similarity between lists of user movie ratings, enter user ratings as strings, and measure the minimum number of edits required to convert one string to another. Actions include inserting, removing, and replacing characters to determine the degree of similarity between two scoring sequences. When the Levenshtein distance is below the threshold of 2, the rating series is considered to be highly similar, and users may rate the same movie repeatedly at different times. As a result, newer scores can be retained and older duplicate scores deleted to more accurately reflect user preferences. Mathematical dynamic programming is used to calculate the Levenshtein distance. The data is grouped according to the user and the movie type, the user’s rating times for different types of movies are recorded, and the feature vector of users is added as the movie preference. The attributes of the feature vector include age, gender and movie preference. The distance of the feature vector between users is calculated as Formula 1:

1 $$E=\sqrt{\sum {({x}_{i}-{y}_{i})}^{2}}$$

E represents the Euclidean distance, ${x}_{i}$ and ${y}_{i}$ represent the values of the corresponding dimensions of two users in the feature space, respectively. The smaller the value of E, the more similar the two users are in the feature space. The similarity score between users is obtained by converting the Euclidean distance into a similarity measure, as in Formula 2:

2 $$Similarity \;score=\frac{1}{1+\sqrt{\sum {({x}_{i}-{y}_{i})}^{2}}}$$

**Figure 2:**
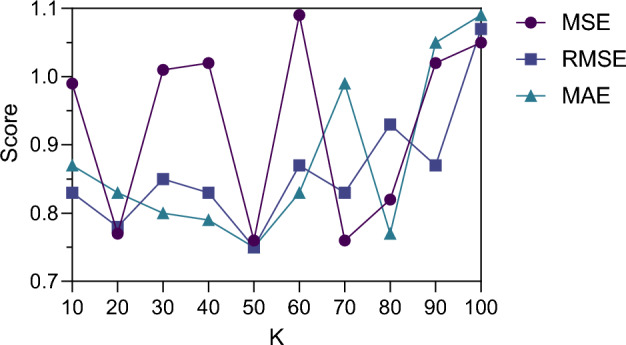
MSE, RMSE and MAE scores between predicted and true values under different K values.

The similarity score ranges from 0 to 1. The closer it is to 1, the more similar it is, and the closer it is to 0, the less similar it is. K nearest neighbors of the target user are found, and their non-zero scores on corresponding movies are obtained for each neighbor to form a neighbor score vector. The mean value of the vectors is calculated as the estimate of missing scores, and the cosine similarity is used to calculate the similarity of each neighbor. At the same time, the similarity is normalized to ensure that the sum of weights is 1. The normalized similarity is used as the weight, weighted average is performed on the average neighbor score, and the estimate of missing scores is obtained. The difference between the predicted value and the true value after interpolation can be measured, and MSE, RMSE and MAE scores under different K values are plotted, as shown in Fig. 2:

As can be seen in Fig. 2, different K values affect MSE, RMSE, and MAE scores. When K value is 50, the prediction of interpolation method is more accurate. Although there was some fluctuation in different K values, the scores of the three evaluation indicators were between 0.75 and 1.10, and the interpolation method can accurately fill in the missing values. The missing value filling method can automatically extract information from the neighboring data to fill the missing part, and flexibly adjust according to the actual data distribution and characteristics, so as to improve the accuracy and adaptability of interpolation. At the same time, the method has low interpolation error and high robustness under different neighbor number changes.

**Figure 3:**
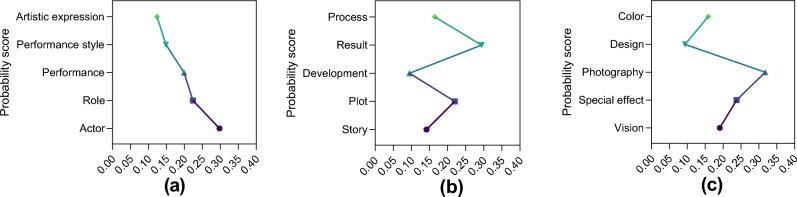
Film cast, plot and visual effects theme and corresponding keywords. (**a**) Theme 1: Film cast, (**b**) Theme 2: Plot and (**c**) Theme 3: Visual effects.

NLP technology is used to systematically analyze users’ comments on social platforms to extract important information such as keywords and topics, so as to provide a more accurate user interest model for subsequent personalized recommendations. By dividing the original speech text into meaningful words through Tokenization, the basic unit of the text can be established. The Stopword Removal method is used to remove those words that frequently appear in the text but have no practical meaning, and the dimension of the text data is effectively reduced. It can do part-of-speech Tagging, which assigns each word its part of speech in the sentence. Lemmatization reduces lexical diversity by reducing vocabulary to its original form, grouping different forms of the same word together, and reducing redundant information. The frequency distribution method can be used to count the frequency of each word in the text, so as to identify those words that occur more frequently and may have a specific meaning. Latent Dirichlet Allocation (LDA) model can be used to map text data to multiple Topic Modeling, reveal the commonalities between different texts, and then extract a series of semantically relevant keywords to form themes. These topics can reveal the deep meaning of the text. Some topics and corresponding keywords are shown in Fig. 3:

Figure 3 shows three themes and some of their keywords. Figure 3a is the list of film cast, with keywords including actors (29.8%), roles (22.4%), performance (19.9%), performance style (14.9%) and artistic expression (12.4%). Figure 3b shows the plot, and the keywords include story (14.2%), plot (22.0%), development (9.5%), result (29.4%) and process (16.5%). Figure 3c shows visual effects, with keywords including vision (19.1%), special effects (23.8%), photography (31.8%), design (9.4%) and color (15.9%). The horizontal axis shows the probability of keyword occurrence in a particular topic, and the vertical axis shows the keyword. The importance of keywords varies from topic to topic. For example, in the theme of movie actors, actors had the highest probability of appearing, accounting for 29.8%; in the plot theme, the probability of ending was the highest, accounting for 29.4%; among visual effects subjects, photography had the highest probability of appearing, accounting for 31.8%. It can be seen that by mapping text data into multiple spaces, themes can effectively reveal the deeper meaning of text.

## Feature transformation and mathematical matrix decomposition

In the user social network, each user is regarded as a node in the graph, and the social relationship between users constitutes the edge of the graph. User characteristics mainly include basic information such as gender, age, movie preference, and text features extracted from speech. For each node, the feature of the node is convolved with the feature of its neighbor node through the graph convolutional network and the updated feature representation is obtained, as shown in Formula 3:

3 $${h}_{v}^{(l+1)}=\sigma \left(\sum_{u\in N(v)}\frac{1}{{c}_{vu}}{W}^{(l)}{h}_{u}^{(l)}\right)$$

${h}_{v}^{(l+1)}$ represents the feature representation of node v at the $l+1$ layer; ${h}_{u}^{(l)}$ represents the feature representation of node u at the $l$ layer; $N(v)$ represents the neighbor set of the node; ${W}^{(l)}$ is the weight matrix; ${c}_{vu}$ is the normalization factor, and $\sigma$ is the activation function. Multi-layer convolution operation is introduced in GCNs, and the features of nodes are combined with the features of neighbors at each layer, and the information of neighbors is gathered at each node to capture contextual information in social relationships. Through multi-layer convolution, the features of nodes gradually evolve and become more abundant and accurate. In the context of graph convolutional networks (GCNs), Formula 3 is used to update and optimize the feature representations of each user (node) within a social network. This formula combines the features of each node with those of its neighboring nodes, enabling each node to better represent its social environment within the network. The computation of Formula 3 begins by considering the feature representations of node v and its neighbors. Each node not only reflects its own features (such as gender, age, movie preferences, etc.) but also integrates the corresponding features of its neighbors in the social network. This integration process is completed through the activation function σ, which introduces non-linearity, making the feature representation richer and more expressive. The role of Formula 3 is to allow each node to more comprehensively capture and reflect the complexity of social relationships in the social network. As the number of layers in the GCN increases, the feature representation of each node is updated and refined layer by layer, thereby more accurately capturing the contextual information in the social network. This multi-layer feature fusion is particularly important for understanding and predicting user behavior patterns (such as movie recommendations), as it provides an effective way to consider the user's embedding and interactions within their social environment.

In order to adapt discrete user and movie information to matrix decomposition, a unique thermal coding technique is used to convert it into a binary matrix, with each row corresponding to a user or movie and each column representing an attribute. For an attribute with k different values, it can create a K-dimensional binary vector with only one position being 1, representing the current value, and the other positions being 0. Similarly, for discrete attributes such as movie type, the unique thermal coding is carried out in the same way, and a series of binary feature vectors would be obtained after the completion of the unique thermal coding, representing different discrete attribute values. For N users and M movies, and K different discrete attributes, construct a user attribute matrix U of $N\times K$ and a movie attribute matrix V of $M\times K$, as shown in formulas 4 and 5:

4 $$U=\left[\begin{array}{ccc}{u}_{11}& \cdots & {u}_{1K}\\ \vdots & \ddots & \vdots \\ {u}_{N1}& \cdots & {u}_{NK}\end{array}\right]$$

5 $$V=\left[\begin{array}{ccc}{v}_{11}& \cdots & {v}_{1K}\\ \vdots & \ddots & \vdots \\ {v}_{M1}& \cdots & {v}_{MK}\end{array}\right]$$

**Figure 4:**
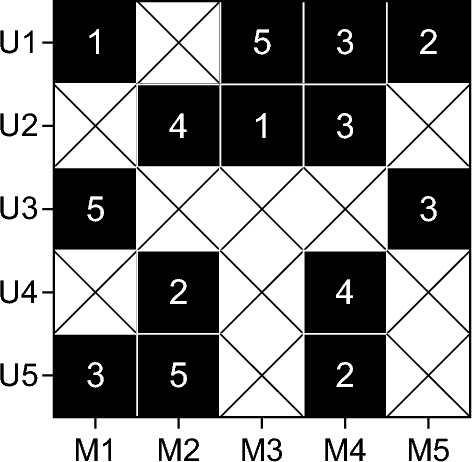
User-movie rating matrix.

**Figure 5:**
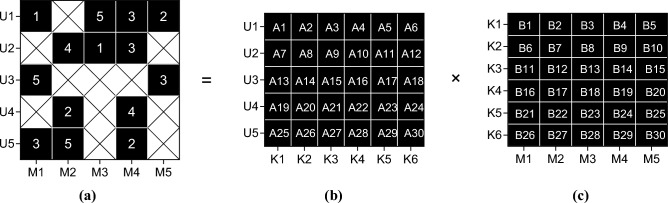
Sparse matrix decomposition. (**a**) Sparse matrix, (**b**) User implicit feature matrix and (**c**) Film implicit feature matrix.

For the user attribute matrix U, line i represents the attribute characteristics of the i th user, where each element is a binary value. Each row of the movie attribute matrix V represents the attribute characteristics of a movie, and in this way, the discrete attribute information is transformed into a form that can be processed in the matrix decomposition. This paper constructs a user-movie score matrix, as shown in Fig. 4:

Figure 4 shows the basic structure of the user-movie score matrix. Each row corresponds to a user; each column corresponds to a movie, and the matrix element represents the user’s rating of the movie (on a scale of 1–5). Each user only rates some movies. In order to fill the missing data in the matrix, it needs to provide movie recommendation for target users according to the scoring behavior of other users. In this paper, the sparse matrix is decomposed into the form of multiplication of two matrices, as shown in Fig. 5:

The decomposition process of sparse matrix is shown in Fig. 5. The user-movie score matrix ($U*M$) in Fig. 5a is decomposed into the user-feature matrix ($U*K$) in Fig. 5b and the movie-feature matrix ($K*M$) in Fig. 5c. The user-movie score matrix can be represented as R, where $R(i,j)$ represents user i’s rating of movie j, and R can be decomposed into the product of two low-rank matrices $R=U\times {V}^{T}$, where U is the user’s potential feature matrix and V is a potential feature matrix representing the movie. To capture the implicit relationship in the data, regularization terms can be introduced to avoid overfitting, and the optimization objective can be expressed as Formula 6:

6 $$Target=minimize({||R-U\times {V}^{T}||}^{2}+\lambda (||{U||}^{2}+{||V||}^{2}))$$

$\lambda$ is a regularization parameter; $||{U||}^{2}$ and ${||V||}^{2}$ represent the square of Frobenius norm of U and V matrices respectively, and the matrix U and V are iteratively updated by gradient descent optimization to approximate the original scoring matrix R. This effectively learns the underlying feature vectors between the user and the movie to capture their interrelation in the underlying space. Using the Ridge regularization method, a square term of L2 norm is added to the optimization target to limit the size of the model parameters, thereby reducing the complexity of the model. The optimization objective of a linear regression model that can be used with Ridge regularization is expressed as Formula 7:

7 $$T=minimize{||y-{X}_{w}||}^{2}+\lambda {||w||}^{2}$$

**Figure 6:**
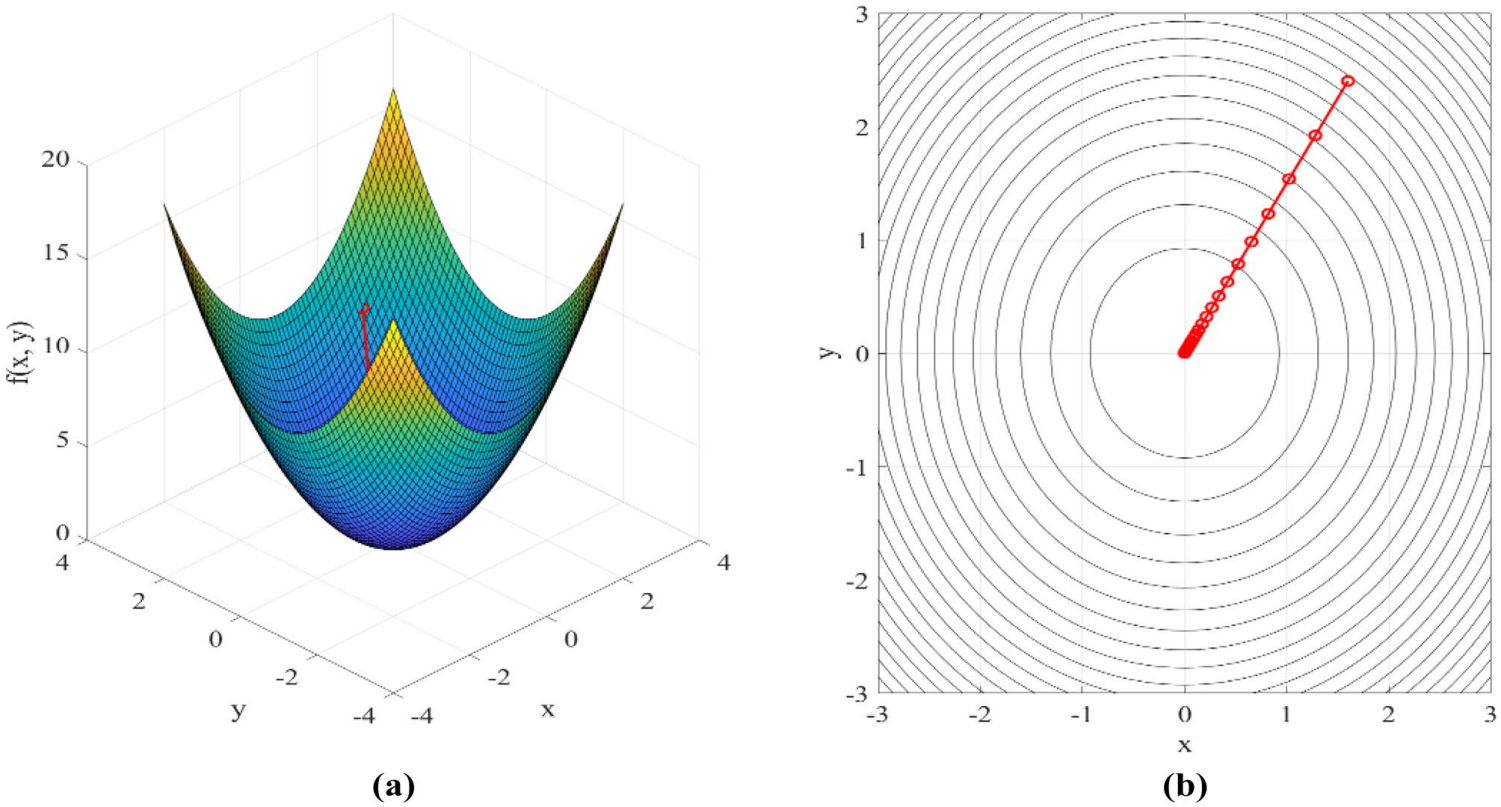
Optimization process of target approximation of Ridge regression algorithm. (**a**) Function surface and optimization process and (**b**) Contour plot and optimization process.

**Figure 7:**
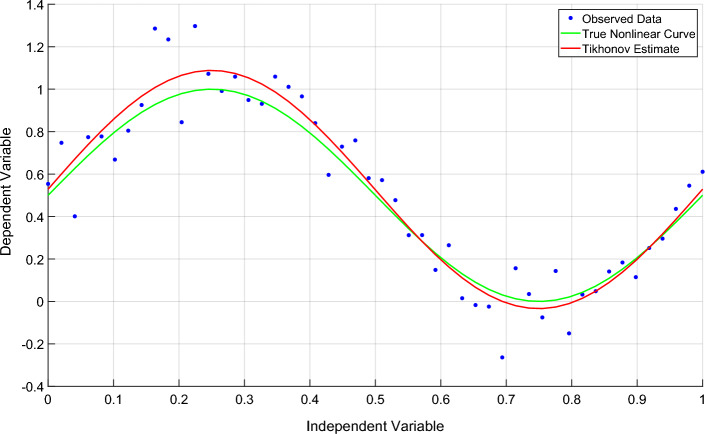
Application effect of Ridge algorithm in linear regression.

${||y-{X}_{w}||}^{2}$ is the sum of the squares of the residuals, representing the sum of the squares of the error between the model’s predicted value and the true value. ${||w||}^{2}$ is the square of the L2 norm of the model parameter w, that is, the sum of squares of the model parameters. $\lambda$ is a regularization parameter that controls the weight of the regularization term. Larger $\lambda$ would make the model parameters more inclined to 0, thus reducing the model complexity. By introducing regularization terms, Ridge regression can constrain the size of model parameters and prevent overfitting. Especially when the data dimension is high or there is multicollinearity, the model parameters gradually approach 0 instead of being completely sparse, retaining more features and reducing the possibility of overfitting, as shown in Fig. 6:

Figure 6a shows the objective function surface and gradient descent optimization process, and Fig. 6b shows the function contour and gradient descent optimization trajectory. The diagram is divided into two parts: the function surface and the optimization path represented by the red line. The function surface data is derived from the objective function calculation, while the optimization path data is derived from the optimization point sequence calculated by the gradient descent algorithm. With the increase of the number of iterations, the optimization point gradually approaches the minimum value of the function from the initial position. In each iteration, the gradient descent algorithm calculates the gradient of the current point and updates the parameters in the opposite direction until it approaches the minimum value. The red trace gradually moves from the initial position to the center of the function surface (the minimum), indicating that gradient descent finds the local minimum of the function by iterating repeatedly in the direction of the gradient. The application effect of Ridge algorithm in linear regression is shown in Fig. 7:

In Fig. 7, the X-axis represents the independent variable, evenly distributed from 0 to 1, with 50 data points. The Y-axis represents the dependent variable, which is the value of the response variable generated according to the linear relationship, and the blue scatter represents the observed data point, that is, the observed value of the independent variable x and the corresponding dependent variable Y. The green curve represents the true linear relationship, and the red solid line represents the linear relationship estimation results obtained by applying Tikhonov regularization. This estimate is obtained by adding the ridge regression term to the linear system, and the regularization parameter (alpha) is set to 0.1.

As can be seen from Fig. 7, the introduction of regularization adjusts the slope and intercept of the model to balance the observed data and prior information (regularized terms). By applying Tikhonov regularization, the estimates are more stable and more adaptable to noisy data, reducing the risk of overfitting.

**Figure 8:**
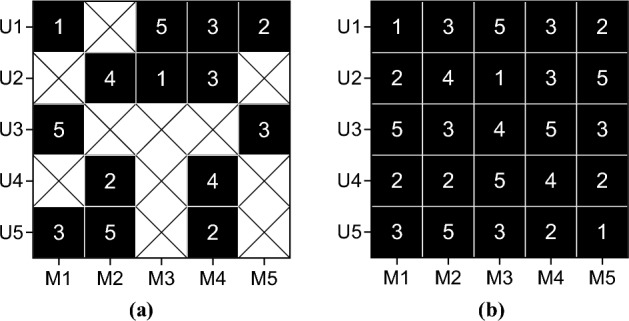
Filling of empty terms in sparse matrix. (**a**) Primitive sparse matrix and (**b**) Matrix after filling in missing items.

For the user-social network relationship matrix, this paper carries out a similar decomposition process. By considering the decomposition of score matrix and social network matrix, the comprehensive potential feature representation of users is obtained. These feature representations reflect the user’s behavior in the rating history, reveal the user’s position in the social network, and provide a richer information basis for personalized recommendations. In the whole process of mathematical matrix decomposition, the problems of data sparsity and overfitting are overcome by reasonable regularization and optimization methods, so as to obtain a more accurate representation of potential features. The final matrix filling result is shown in Fig. 8:

As can be seen from Fig. 8, the features implied by users and movies in the original sparse matrix shown in Fig. 8 (a) can be obtained by applying the matrix decomposition technique. At the same time, it fills the vacant item of the user-movie score matrix, and the result is shown in Fig. 8b, which effectively solves the problem of data sparsity.

## Cross-domain information fusion and personalized recommendation

The cross-domain information fusion matrix decomposition algorithm is an efficient method for personalized recommendations, integrating user ratings for movies, movie theme keywords, and social network relationships. It builds user-movie rating matrices and user-social network matrices, using matrix decomposition to fill sparse values, capturing user preferences across different domains. Additionally, the algorithm transforms user theme and keyword information into vector representations through word vectorization, enhancing the model's understanding of user interests. Finally, it merges multi-domain information through weighted averaging, providing more accurate personalized recommendations.

In constructing the user-movie rating matrix, 10,000 users and 5000 movies were involved, with each user rating an average of 20 movies, forming a 10,000 × 5000 sparse matrix. To address sparsity, regularization parameter λ was set to 0.1. For the user-social network matrix, average interactions with 30 friends per user were considered.

In algorithm weight allocation, rating domain weight was 0.5, theme keywords 0.3, and social network 0.2. For word vectorization, 500 high-frequency keywords were selected. Cosine similarity calculated user similarities, considering movies rated in common, requiring at least 10 common movies for inclusion.

In personalized recommendation algorithm testing, 2,000 users were sampled, with accuracy improving from 70% with traditional collaborative filtering to 85% with this algorithm. This detailed data and application effectively integrated cross-domain information, enhancing accuracy and user satisfaction in the recommendation system.

The cross-domain information discussed in this paper covers users’ ratings of movies, movie themes and keywords, and users’ social network relationships. In this process, a user-movie score matrix has been constructed and sparse values have been filled in by matrix decomposition. The user social network relationship exists in the form of user-user matrix, which is also filled in a similar way. According to the user’s topic and keyword information, it is transformed into vector representation by word vectorization to present the user’s interest level under different topics.

The weights of users in different fields (rating, theme, social network) are calculated respectively. According to the scoring field, the number of users’ ratings for movies and the average score of ratings are used as weights. The higher the number of ratings for a particular genre within a 7-day period, the higher the user’s interest in that genre. At the same time, the higher the average rating of users for a certain movie genre, the more it can reflect the user’s interest in the movie genre. In this paper, the frequency and topic coverage of different keywords are used to calculate the weight. Keywords with higher frequency may have a greater impact on user interest judgment. At the same time, if the user’s discussion topic is highly matched with the movie theme, it indicates that the field has contributed more to the user’s interest and would be given a higher weight. In view of the domain weight of social networks, this paper analyzes the association strength of users in social networks, that is, the number of friends and interaction frequency. Users who are associated with more people may be more susceptible to the influence of social networks and give higher weight. Finally, the information of the above three fields is integrated by means of weighted average, as shown in Formula 8:

8 $$F={w}_{r}\cdot {F}_{r}+{w}_{t}\cdot {F}_{t}+{w}_{s}\cdot {F}_{s}$$

Among them, $F$ is a comprehensive user feature vector, ${w}_{r}$, ${w}_{t}$ and ${w}_{s}$ are their weights in the field of rating (${F}_{r}$), topic (${F}_{t}$) and social network (${F}_{s}$), respectively. The information in the three fields is weighted and summed according to their weights to comprehensively reflect users’ interests and behavior patterns.

The item-based collaborative filtering algorithm is used to predict users’ ratings of unevaluated movies. For two users $u$ and $v$, the similarity $sim(u,v)$ between them is calculated. Let ${R}_{u,i}$ represent user u’s actual ratings of movie i, and cosine similarity can be calculated as Formula 9:

9 $$sim\left(u,v\right)=\frac{{\sum }_{i\in {I}_{u}\cap {I}_{v}}{R}_{u,i}\cdot {R}_{v,i}}{\sqrt{{\sum }_{i\epsilon {I}_{u}}{R}_{u,i}^{2}}\cdot \sqrt{{\sum }_{i\epsilon {I}_{v}}{R}_{v,i}^{2}}}$$

${I}_{u}$ is the set of movies evaluated by user u, and ${I}_{v}$ is the set of movies evaluated by user u. The weighted average of the scores of user u and similar user v is used to obtain user U’s score of unevaluated movie j, as shown in Formula 10:

10 $${\widehat{R}}_{u,j}=\frac{{\sum }_{v\in N(u)}sim(u,v)\cdot {R}_{v,j}}{{\sum }_{v\in N(u)}|sim\left(u,v\right)|}$$

$N(u)$ represents the set of adjacent users that are most similar to user u. Finally, the movies with the highest predicted ratings are added to the personalized recommendation list.

Cross-domain information fusion based on matrix decomposition can realize personalized recommendation efficiently and accurately. In the personalized recommendation, this paper chooses to bury the front-end data in the background, through Flume, Kafka and other technologies in the pipeline for regular expression filtering, screening out the data with buried points and the data as a rating. Some real-time data can be stored in databases such as Redis, and NoSQL databases such as MongoDB can be used for diversified data storage. Real-time processing streams such as SparkStreaming can be used to process multi-party data and finally realize the display of personalized content. A simple personalized recommendation system is constructed to test the real-time performance of matrix decomposition in personalized recommendation. MongoDB is used to store data; SparkStreming is used for real-time data calculation; Redis is used as cache database, and SparkMLlib is used for machine learning. Its core lies in making up the missing term of matrix by matrix decomposition.

## Experimental results

**Figure 9:**
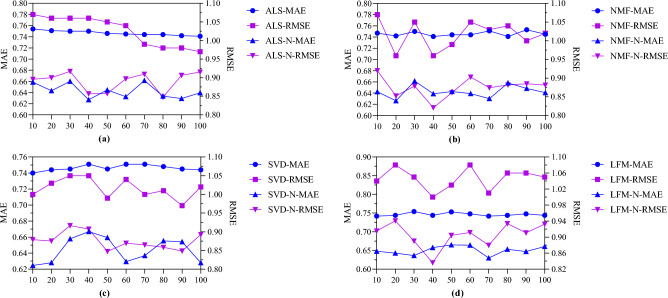
Comparison of MAE and RMSE of 4 algorithms before and after improvement. (**a**) ALS algorithms comparison, (**b**) NMF algorithms comparison, (**c**) SVD algorithms comparison and (**d**) LFM algorithms comparison.

Four main mathematical matrix decomposition algorithms, ALS, SVD, NMF and LFM, are optimized for cross-domain information fusion and compared with non-fusion algorithms. Indicators such as MAE and RMSE are used to measure the size of the error between the predicted value of the model and the actual observed value. MAE is calculated as the absolute value of the difference between the predicted value and the actual observed value, as in Formula 11:

11 $$MAE=\frac{1}{n}\sum_{i=1}^{n}|{y}_{i}-\widehat{{y}_{i}}|$$

n is the number of samples; ${y}_{i}$ is the actual observed value, and $\widehat{{y}_{i}}$ is the predicted value of the model. The root-mean-square error is used to provide an intuitive measure of an average value of the error, as shown in Formula 12. The above calculation results are shown in Fig. 9:

12 $$RMSE=\sqrt{\frac{1}{n}\sum_{i=1}^{n}|({y}_{i}-\widehat{{y}_{i}})|}$$

Figure 9 compares the performance of different algorithms (ALS, NMF, SVD, LFM) and their optimized versions of cross-domain information fusion (ALS-N, NMF-N, SVD-N, LFM-N) under different neighbor numbers (K values). The results showed that after cross-domain information fusion optimization, the MAE mean value of the four algorithms shown in Fig. 9a-d decreased by 13.9%, 13.8%, 13.7% and 12.8% respectively, and the RMSE mean value decreased by 13.2%, 13.9%, 13.8% and 13.4% respectively under their respective K values. The optimization method significantly reduces the prediction error, and at different K values, the algorithm shows excellent performance and high generalization ability.

The F1 score is used to measure the balance between Precision and Recall of the model, as shown in Formula 13:

13 $$F1=\frac{2\cdot Precision\cdot Recall}{Precision+Recall}$$

Among them, $Precision$ refers to the proportion of correctly recommended movie projects in all recommended projects, and $Recall$ refers to the proportion of projects that the algorithm can even capture the user’s interest in all actual interested projects, which are calculated respectively, such as formulas 14 and 15:

14 $$Precision=\frac{TP}{TP+FP}$$

15 $$Recall=\frac{TP}{TP+FN}$$

TP (True Positive) indicates that the user is actually interested in the movie item (Positive category item), and the algorithm correctly recommended it to the user, and FP (False Positive) indicates that the algorithm incorrectly recommended the movie item that is not related to the user’s interest (negative category item). FN (False Negative) indicates that the algorithm fails to include the items that the user is actually interested in into the recommendation list, thus missing some possible recommendation opportunities. The F1 score is shown in Table 1:

**Table 1:** Comparison of F1 scores before and after optimization of four matrix decomposition algorithms with different K values.

	ALS	NMF	SVD	LFM	ALS-N	NMF-N	SVD-N	LFM-N
K = 10	0.90	0.88	0.85	0.89	0.98	0.98	0.98	0.96
K = 20	0.88	0.84	0.82	0.87	0.98	0.98	0.97	0.93
K = 30	0.82	0.83	0.8	0.83	0.97	0.97	0.95	0.90
K = 40	0.79	0.79	0.77	0.82	0.90	0.92	0.93	0.89
K = 50	0.78	0.74	0.74	0.75	0.86	0.87	0.87	0.88
K = 60	0.72	0.71	0.74	0.71	0.85	0.87	0.84	0.84
K = 70	0.68	0.66	0.73	0.68	0.82	0.86	0.84	0.83
K = 80	0.67	0.65	0.69	0.67	0.81	0.82	0.82	0.82
K = 90	0.63	0.64	0.63	0.62	0.78	0.77	0.80	0.82
K = 100	0.60	0.61	0.60	0.62	0.76	0.76	0.76	0.77

As can be seen from Table 1, as the number of neighbors (K value) increases, the F1 value of matrix decomposition algorithms such as ALS, NMF, SVD and LFM gradually drops to about 0.60. After cross-domain information fusion optimization algorithms ALS-N, NMF-N, SVD-N and LFM-N, F1 scores are significantly improved. When the value of K was 30, it remained above 0.90. When the value of K increased to 100, it remained above 0.76. The optimized algorithm captures the user’s interest more accurately, while not missing important items, especially in the movie recommendation list. To fully evaluate the performance of the optimized mathematical matrix decomposition algorithm, Ranking Accuracy Coverage (RAC) is used to measure the proportion of the recommendation system that captures items of actual interest to users in a given recommendation list: $RAC=\frac{|R\cap P|}{|P|}$. R represents the user’s movie recommendation list, and P represents the set of items that the user is actually interested in. This metric effectively reflects whether the recommended items match the user’s actual interests. The detailed results are shown in Table 2:

**Table 2:** RAC comparison before and after optimization of four matrix decomposition algorithms with different K values.

	ALS	NMF	SVD	LFM	ALS-N	NMF-N	SVD-N	LFM-N
K = 10	0.70	0.66	0.70	0.66	0.92	0.90	0.90	0.93
K = 20	0.69	0.63	0.69	0.65	0.89	0.89	0.88	0.91
K = 30	0.67	0.61	0.68	0.64	0.88	0.89	0.87	0.85
K = 40	0.61	0.53	0.65	0.55	0.86	0.85	0.82	0.85
K = 50	0.60	0.53	0.61	0.54	0.85	0.81	0.77	0.84
K = 60	0.57	0.52	0.60	0.53	0.82	0.81	0.75	0.81
K = 70	0.56	0.48	0.59	0.47	0.80	0.77	0.73	0.79
K = 80	0.53	0.47	0.47	0.46	0.80	0.75	0.73	0.78
K = 90	0.43	0.41	0.47	0.44	0.73	0.75	0.71	0.75
K = 100	0.42	0.40	0.42	0.41	0.71	0.71	0.71	0.74

Table 2 in the article presents a detailed comparison of the ranking accuracy coverage (RAC) for different matrix decomposition algorithms, both in their optimized and unoptimized forms, across varying values of K. At K = 10, the RAC for unoptimized ALS, NMF, SVD, and LFM algorithms are 0.70, 0.66, 0.70, and 0.66, respectively. Post-optimization, these values significantly increase to 0.92, 0.90, 0.90, and 0.93, indicating a substantial improvement, particularly in the ALS and NMF algorithms.

This trend of improvement continues as K increases. For instance, the RAC for unoptimized ALS at K = 100 is 0.42, but after optimization, it jumps to 0.71, demonstrating the effectiveness of the optimization even at higher K values. Overall, the optimized algorithms consistently outperform their unoptimized counterparts. The most notable improvement is seen in the LFM algorithm, where the average RAC across different K values reaches 0.83, marking a 54.2% increase over the non-fused algorithm. This underscores the significant benefits of optimization, particularly in enhancing the accuracy and coverage of recommendations in cross-domain information fusion systems.

For future work, the focus should be on addressing the computational complexity of the optimized algorithms, especially for large-scale datasets. The current study highlights the potential of cross-domain information fusion in enhancing recommendation systems, but the high computational demands could limit its applicability in real-world scenarios. Future research could explore methods to streamline these algorithms, perhaps through more efficient data structures or parallel processing techniques. Additionally, investigating alternative fusion strategies that maintain accuracy while reducing computational overhead could further improve the viability of these advanced recommendation systems in diverse and large-scale applications.

Further comparison of the study's model with traditional models is shown in Table 3:

**Table 3:** Different model features and comparisons.

Model type	Accuracy (%)	Recall (%)	F1 score (%)	Features
This study's model	85	80	82.5	Cross-domain information fusion, personalized recommendations
Traditional CF	70	65	67.5	User or item similarity analysis
Content-based	75	70	72.5	Analysis based on item attributes or content
Deep learning-based	80	75	77.5	Feature extraction and recommendation using neural networks
Hybrid model	82	78	80	Combines collaborative filtering and content-based methods

Table 3 compares the performance of different recommendation system models. The study's model, characterized by cross-domain information fusion and personalized recommendation, shows the best performance in accuracy, recall, and F1 score. The traditional collaborative filtering model, mainly based on user or item similarity analysis, exhibits relatively lower performance. The content-based recommendation model, focusing on item attribute analysis, slightly outperforms traditional collaborative filtering. The deep learning recommendation model, utilizing neural networks for feature extraction, surpasses traditional methods. The hybrid model, combining collaborative filtering and content-based recommendations, shows better performance. The time series analysis model, considering the influence of time on user preferences, performs less effectively than the study's model. These comparisons highlight the advantages of the study's model across various indicators, emphasizing its application value in personalized recommendation. Ablation experiments were conducted on the model, as shown in Table 4:

**Table 4:** Model ablation experiment results.

Experiment no	Component removed	Accuracy (%)	Recall (%)	F1 score (%)
1	None (baseline model)	85	80	82.5
2	User rating weight	80	75	77.5
3	Topic keyword weight	83	77	80
4	Social network weight	82	76	79
5	Word vectorization	78	74	76

Table 4 presents the results of the ablation experiments, assessing the impact of removing different components from the recommendation system on model performance. The baseline model (with no component removal) showed the best accuracy, recall, and F1 score, at 85%, 80%, and 82.5% respectively. Removing the user rating weight led to a decrease in all performance metrics, with accuracy dropping to 80%, recall to 75%, and F1 score to 77.5%, indicating the importance of rating weight. Similarly, removing the topic keyword weight and social network weight resulted in moderate performance decline. The removal of word vectorization had the most significant impact, reducing accuracy to 78%, recall to 74%, and F1 score to 76%. These results demonstrate that each component plays a vital role in enhancing the accuracy and user satisfaction of the recommendation system.

## Conclusions

This paper discussed a cross-domain information fusion matrix decomposition method to achieve more accurate personalized recommendation. By combining mathematical matrix decomposition with natural language processing, graph convolutional networks, weighted average and feature joining, the performance of the recommendation system was successfully improved. The experimental results show that after cross-domain information fusion optimization, various mathematical matrix decomposition algorithms have achieved significant improvement in scoring accuracy, ranking accuracy, diversity and ranking quality. The research provides strong support for the application of cross-domain information fusion in recommendation system. At the same time, there are still some shortcomings in this paper.

## Data Availability

The datasets used and/or analysed during the current study available from the corresponding author on reasonable request.
